# Efficacy of Hydroponically Cultivated Saffron in the Preservation of Retinal Pigment Epithelium

**DOI:** 10.3390/molecules28041699

**Published:** 2023-02-10

**Authors:** Mattia Di Paolo, Francesca Corsi, Maria Maggi, Luca Nardi, Silvia Bisti, Ilaria Piano, Claudia Gargini

**Affiliations:** 1 Department of Ophthalmology and Visual Sciences, University of Louisville, Kentucky, KY 40202, USA; 2 Department of Pharmacy, University of Pisa, 56126 Pisa, Italy; 3 National Institute of Biostructure and Biosystem (INBB), V. le Medaglie D’Oro 305, 00136 Roma, Italy; 4 Hortus Novus srl, via Campo Sportivo 2, 67050 Canistro, Italy; 5 Department of Physical and Chemical Sciences, University of L’Aquila, 67100 Coppito, Italy; 6 ENEA, Italian National Agency for New Technologies, Energy and Sustainable Economic Development, Biotechnology and Agro-Industry Division, Casaccia Research Center, 00123 Rome, Italy

**Keywords:** REPRON^®^, retinal disease, ARPE-19, hydroponic

## Abstract

Saffron treatment is a broad-spectrum therapy used for several retinal diseases, and its effectiveness depends on a particular molecular composition (REPRON^®^ saffron). Its production requires specific crops and procedures that, together with low yields, make this spice expensive. To reduce costs, the use of hydroponic crops is gradually increasing. In this study, we tested the protective properties of a hydroponic saffron (sH) batch in models of retinal pigmented epithelium (RPE) degeneration. ARPE-19 cells were pretreated with 40 µg/mL saffron and exposed to different types of damage: excess light and retinol (LE + RET) or oxidative stress (H_2_O_2_). After analyzing the composition of all saffron types with spectroscopy, we performed cell viability and immunofluorescence analysis for both protocols. We compared the sH results with those of a validated batch of saffron REPRON^®^ (sR) and those of a saffron non-REPRON^®^ (sNR) batch. sH and sR, which we found had the same chemical composition, were more effective than sNR in increasing cell survival and attenuating the morphological changes related to apoptosis. In conclusion, hydroponic culturing is a suitable strategy to produce high-quality saffron to reduce costs and increase the accessibility of this promising treatment for retinal degeneration.

## 1. Introduction

Saffron treatment is a promising therapy for several types of neurodegeneration; moreover, it is particularly effective in retinal diseases [1]. In the last 20 years, interest in the use of saffron for treating neurodegenerative processes has substantially increased, which may predict an increasing demand for this spice for clinical use. The chemical composition of saffron is complex: this spice has primary metabolites that are ubiquitous in nature, such as carbohydrates, minerals, fats, vitamins, amino acids, and proteins, as well as many compounds belonging to different classes of secondary metabolites, such as carotenoids, monoterpenes, and flavonoids, especially including anthocyanins [2].

Among the secondary metabolites, the most important are the carotenoids, which are responsible for the color of the spice. The carotenoids include fat-soluble ones, such as α- and β-carotene, lycopene, and zeaxanthin, as well as water-soluble ones, such as apocarotenoid crocetin (C_20_H_24_O_4_) and crocins, and the polyene esters of the mono- and diglycoside crocetin.

Crocins comprise 3.5% of the weight of a plant’s stigmas. Saffron glycoside carotenoids, as with all glycosides, are usually thermally labile and photochemically sensitive, especially in solution. As with crocetin, crocins exist in two isomeric forms: 13-cis and all-trans. When discussing crocins, we are referring to a large family of compounds, because several carbohydrates can esterify one or both of the carboxyl groups of crocetin in its different isomeric forms. These substances, which differ only in configuration and substituents, have similar physicochemical properties, especially polarity. These similarities hinder their chemical characterization (separation, identification, etc.) [3,4,5,6].

Two compounds are obtained from the oxidation of the carotenoids picrocrocin (monoterpene glycoside) and safranal (cyclic monoterpene aldehyde), which are responsible for the bittering and aromatic strength of the spice, respectively. According to the most accredited hypothesis, zeaxanthin is the precursor of these two substances. The enzyme responsible for the transformation of zeaxanthin to crocetin dialdehyde is crocus zeaxanthin 7,8(7,8)-cleavage dioxygenase (CsZCD) [7]; the latter can be oxidized and esterified by several glucosyltransferases to produce crocins [8] and picrocrocin. Picrocrocin (C_16_H_26_O_7_), which constitutes 3.7% of the weight of the stigma, is found only in the genus *Crocus*, of which the only edible spice is *Crocus sativus* L.; therefore, it constitutes a molecular marker of saffron. During the drying process, the enzyme β-glucosidase acts on picrocrocin to release 4-hydroxy-2,6,6-trimethyl-1-cyclohexene-1-carboxaldehyde (HTCC, C_10_H_16_O_2_) [9], which, by dehydration, is transformed into safranal (C_10_H_14_O). This is present at 0.02% in the stigma and is the main representative of the volatile fraction of saffron.

The chemical composition of saffron is strictly correlated with some of the properties of this spice [10]. The chemical analyses (qualitative and quantitative) of many saffron samples, conducted in parallel with the treatment of animal models, showed that the neuroprotective activity of this spice depends on the content of some of its active compounds, in particular, on the concentration of the two most abundant crocins (trans-crocetin bis (β-d-gentiobiosyl) ester (T1) and trans-crocetin (β-d-gentiobiosyl, β-d-glucosyl) ester (T2)). These results led to the filing of an international patent for a saffron product (REPRON^®^) [11].

Hydroponic culture might represent an innovative system to maximize and optimize the yield of saffron [12]. However, whether this mode of production affects the spice quality and its cell-protective effect is not fully understood. To address this issue, in this study, we analyzed all samples obtained from hydroponic cultivation using different analytical methods to characterize their chemical composition. We compared the results with those from a sample of saffron cultivated according to the traditional method (in the field). Subsequently, we developed degenerative in vitro models of RPE cells for saffron screening. The majority of retinal diseases, such as retinitis pigmentosa (RP) [13] (RP) or age-related macular degeneration (AMD) [14], might affect several RPE-dependent processes, such as the visual cycle. Impairments in photo-pigment recycling might lead to the abnormal accumulation of molecule intermediates, which induce oxidative stress and apoptosis [15]. As the literature suggests, saffron treatment improves ARPE-19 cells’ basal metabolism [1]. In this study, we tested saffron mechanisms with two different in vitro models of degenerative RPE, induced either by excess retinol and light or by oxidative stress. Here, we provide evidence of the cell-protective properties of a batch of saffron produced via hydroponic culture, which are similar to those obtained with spice produced using the classic horticulture method. The analyzed chemical composition confirmed the ratio among molecules, which meets the REPRON^®^ classification requirement.

## 2. Results

### 2.1. Chemical Composition of Saffron Samples

For each sample of saffron, we spectrophotometrically analyzed an aqueous extract of the spice, according to ISO 3632 [9], to determine the class of each sample and, therefore, its market value. From the absorbance values at three different wavelengths (440, 330, and 257 nm), obtained from the UV–Vis absorption spectrum of the aqueous extract of saffron, we determined three values: E440: coloring power, 440 nm absorption crocin; E257: bittering power, 257 nm absorption picrocrocrocin; E330: aromatic power, 330 nm absorption safranal.

**Figure 1:**
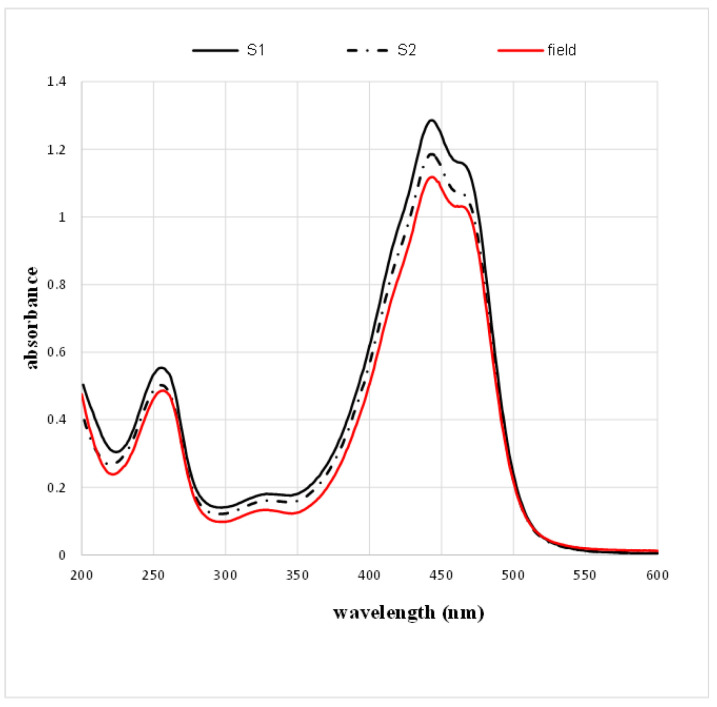
Absorption spectra of saffron samples. S1 and S2 are hydroponic saffron grown in a greenhouse and in the field using the traditional method, respectively.

By comparing the values obtained with those provided by the ISO standard, we determined the product class of each sample. The calculated values refer to the dry weight of saffron; therefore, another parameter to be determined was moisture. The absorption spectra of the samples are reported in Figure 1.

Figure 1 shows that the absolute maximum of the spectra (absorbance at 440 nm) is greater than or equal to one, indicating that all samples were class I, in accordance with the ISO standard.

Table 1 reports the coloring, bittering, and aromatic power of the samples. The values reported in Table 1 confirm what was predicted by the spectra: all samples were of excellent quality (class I), and we found high homogeneity in the values of humidity, which showed how the drying process of stigmas is reproducible.

After the spectrophotometric analysis, we performed a chromatographic analysis on a hydro-alcoholic extract of the samples [16]. We used chromatographic analysis to determine the concentration (%) of trans-4 gentobiose-gentobiose crocin (T1) and trans-3 gentobiose-glucose crocins (T2); the percent concentrations of the two crocins refer to milligrams of crocin per 100 g of dry saffron [17]. Based on the concentrations of the two crocins, we could classify samples as REPRON^®^ or not. If a sample of saffron was REPRON^®^, its neuroprotective activity was confirmed, and its ability to be used in the ophthalmic field was verified.

The determination of the concentration of the two crocins was only possible for samples for which the coloring power was determined (Table 1), because the concentration is calculated as follows [17]:

(1) $$c\left(\mathrm{mg}/g\right)=\frac{MW_{i}\times E_{1\mathrm{cm}}^{1\%}\left(440\;\mathrm{nm}\right)\times A_{i}}{\varepsilon_{t,c}}$$

where $MW_{i}$ and $A_{i}$ are the molecular weight and the percentage peak area, respectively; $E_{1\mathrm{cm}}^{1\%}\left(440\;\mathrm{nm}\right)$ is the coloring strength of the saffron sample and $\varepsilon_{t,c}$ is the extinction coefficient (89.000 M^−1^ cm^−1^ for 215 trans-crocins and 63.350 M^−1^ cm^−1^ for cis-crocins).

**Figure 2:**
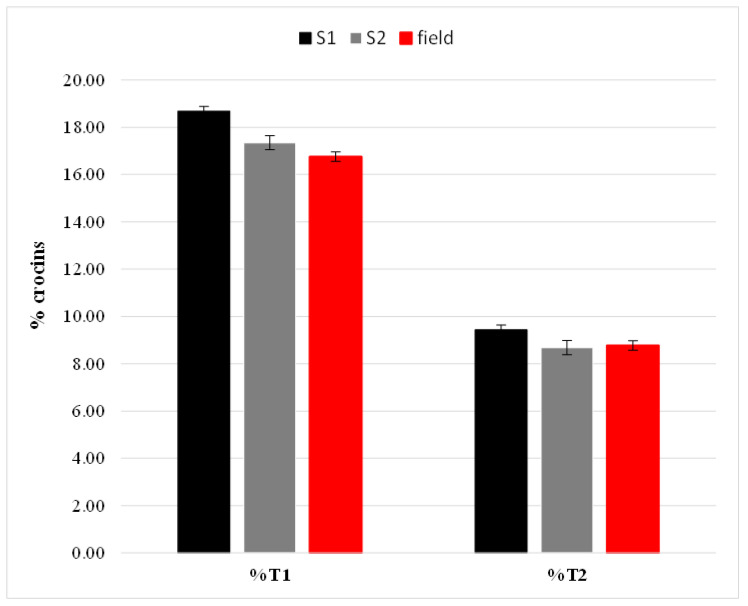
Percentage (%) of crocins (trans-4 gentobiose-gentobiose crocin (T1) and trans-3 gentobiose-glucose crocins (T2)) in S1 and S2 (hydroponic saffron grown in greenhouse and saffron grown in field, respectively).

We performed chromatographic analysis in triplicate on each sample to assess the robustness and reproducibility of the method. From the percent values (milligrams of crocin in 100 g of dry saffron), we deduced that all three analyzed samples were REPRON^®^, and we found no statistically significant differences in the contents of the two crocins. The results are shown in Figure 2.

### 2.2. Saffron Treatment Improves Cell Viability and Preserves Cell Morphology in Retinol + Light-Induced RPE Degeneration

**Figure 3:**
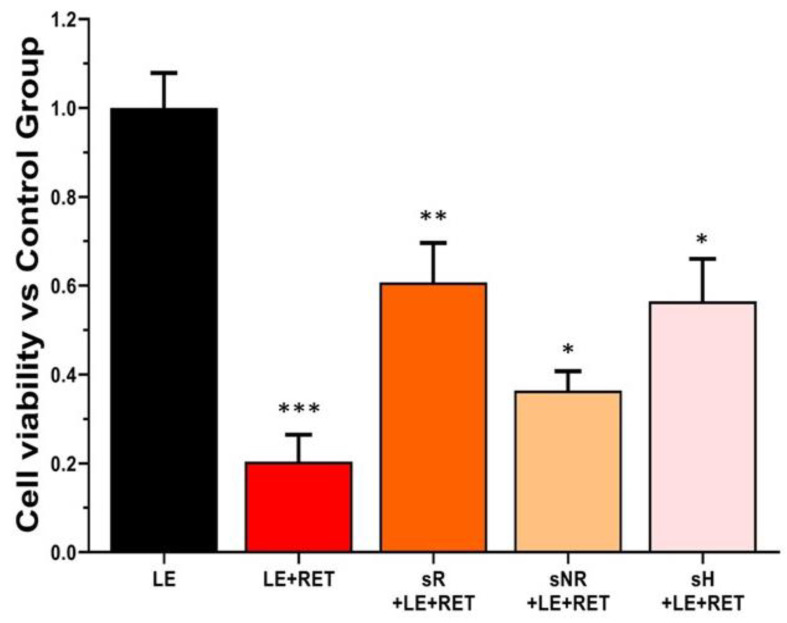
Cell survival test. Normalized cell viability assay of ARPE-19 cells exposed to retinol (xhalf: 5.6 M) and different types of saffron. Hydroponic saffron treatments (sH) significantly increased the survival rate. REPRON^®^ saffron (sR) had the strongest protective effect, whereas non-REPRON^®^ saffron (sNR) did not notably preserve cells from retinol toxicity. LE, light exposure; LE + RET, light exposure in presence of retinol; LE + RET + sR, light exposure in presence of retinol and saffron REPRON^®^; LE + RET + sNR, light exposure in presence of retinol and saffron non-REPRON^®^; LE + RET + sH, light exposure in presence of retinol and hydroponic ENEA saffron. Values in the graph indicate cell viability as mean ± SE (*n* = 3). One-way analysis of variance (ANOVA) test followed by Dunnett’s test, * *p* > 0.05 (sNR vs. LE + RET; sH vs. LE + RET); ** *p* ≤ 0.01 (sR vs. LE + RET); *** *p* ≤ 0.001 (LE vs. LE + RET).

The beneficial effects of saffron on the basal metabolism of ARPE-19 cells [1] suggest that saffron can protect cells, ensuring survival from low retinol toxicity. We tested different batches of saffron following the experimental protocol, as shown in Figure 7. In particular, two types of high-quality saffron [18], sR and sH, remarkably preserved cell survival, whereas a saffron lot with a different composition, sNR, did not positively affect cell viability (Figure 3).

**Figure 4:**
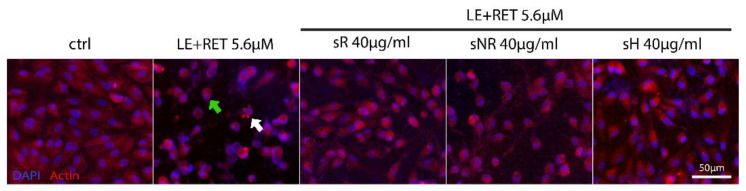
Preservation of ARPE-19 cell morphology in degenerative model induced via LE + RET exposure. Representative images of cytoskeleton (actin, red) and nucleus (DAPI, blue) in all experimental groups. Exposure to retinol and light produced cellular shrinkage with cytoskeleton and nuclei condensation (LE + RET, actin, red, white arrow; DAPI, blue, white arrow). Treatment with saffron, i.e., with sR and sH, was more effective in preserving cell morphology, whereas the cell shape was similar to that of the control (LE). Scale bar = 50 µm.

The corresponding immunofluorescence in Figure 4 describes the morphological profile of each experimental group. Cells exposed to LE + RET showed signs of stress, with reduced volume and a condensed cytoskeleton [19] (Figure 4, actin, red; white arrow). In addition, some of them showed a peripheral nucleus [20] (Figure 4, 4′,6-diamidino-2-phenylindole (DAPI), blue; green arrow). We observed similar features in all treatments with saffron; however, compared to the LE + RET group, cellular shape was better preserved in sR and sH.

### 2.3. Saffron Treatment Improves Cell Viability and Gap Junction Integrity in H_2_O_2_-Induced RPE Degeneration

**Figure 5:**
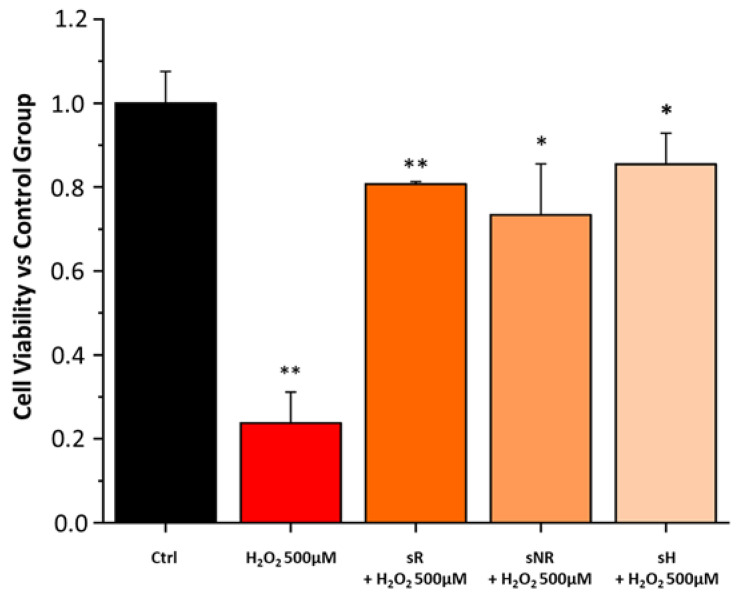
Cell survival test. Normalized cell viability assay of ARPE-19 cells exposed to H_2_O_2_ and different types of saffron. Pretreatment with saffron significantly decreased cell death due to damage with H_2_O_2_. Cell viability was analyzed using CellTiter 96 Aqueous One Solution reagent. Cells were pretreated for 24 h with the various saffron samples (REPRON saffron, sR; non-REPRON saffron, sNR; hydroponic saffron, sH) at 40 μg/ml and then exposed for 3 h with H_2_O_2_ 500 μM. Cell viability was normalized to Ctrl: no pretreatment and no H_2_O_2_ exposure (black bar). Values in the graph indicate cell viability as the mean ± SE obtained from *n* = 3 of independent experiments; Statistics: One-way ANOVA test followed by Levene’s post-test. * *p* ≤ 0.05 (sNR vs. H_2_O_2_ 500 μM; sH vs. H_2_O_2_ 500 μM); ** *p* ≤ 0.01 (Ctrl vs. H_2_O_2_ 500 μM; sR vs. H_2_O_2_ 500 μM).

We evaluated the efficacy of the various saffron samples (REPRON^®^ saffron (sR); non-REPRON^®^ saffron (sNR); hydroponic saffron (sH)) in preserving cell viability in a cell model of ARPE-19 with H_2_O_2_-induced stress. All saffron samples were able to decrease cell death due to H_2_O_2_-stress and preserve cell viability (Figure 5).

**Figure 6:**
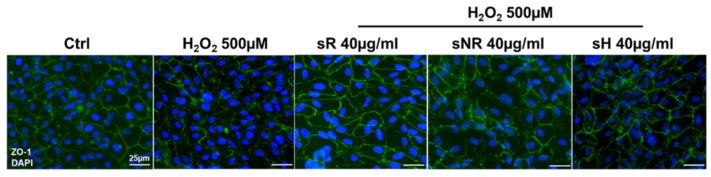
Preservation of tight junction morphology from H_2_O_2_-induced oxidative damage in the ARPE-19 cell model. Panel shows the marking for the tight-junction-specific protein zonula occludens 1 (ZO-1, green) in control cells (no pretreatment and no H_2_O_2_ exposure), in cells damaged by 500 µM H_2_O_2_ for 3 h (H_2_O_2_ 500 µM), and in cells pretreated with 40 µg/mL of the various saffron samples (REPRON^®^ saffron (sR); non-REPRON^®^ saffron (sNR); hydroponic saffron (sH)). Nuclei were counterstained with DAPI. Scale bars = 25 µm.

To validate the protective function of saffron, we studied the preservation of tight junction morphology using the immunofluorescence of the tight-junction-specific protein zonula occludens 1 (ZO-1). From the images, we observed how pretreatment with sR and sH preserved the integrity of the junctions, preserving the cell structure similar to that found in the control cells. This protective activity seemed to be maintained, albeit to a lesser degree, by sNR. In the image, at several points, the junctions seem to be destroyed (Figure 6).

## 3. Discussion

Saffron treatment is a therapy already used for several retinal neurodegeneration diseases such as AMD and Stargardt’s disease [21,22]. Its effectiveness is not completely understood; however, it may depend on a specific molecular composition, leading to a synergistic action. Experimental results from previous studies [10,11] have emphasized that chemical composition is critical for saffron efficacy in neuroprotection, and all the components appear essential to provide the best protection [1,10]. Altogether, all the data obtained confirmed the idea that natural compounds that contain many molecules must be simultaneously tested from different aspects to precisely define the chemical characteristics of the active compound to optimize treatment and obtain better and more reproducible results. Until now, the relationship between chemical composition and neuroprotective activity was only demonstrated for field-grown saffron; in this study, we tested the hypothesis that saffron grown using innovative methods, such as hydroponic cultivation, might have the same chemical characteristics as field-grown saffron, and can therefore be labeled as REPRON^®^. Cultivating saffron in a controlled environment presents several advantages, including the possibility of producing pharma-grade products. Previously, to define REPRON^®^ characterization, parallel experiments were performed, namely, saffron treatment in neuro-degenerating retinas and an analytical chemical analysis of the saffron used [10]. Therefore, standardized hydroponic cultures were recently developed to guarantee the consistent production of high-quality saffron. Here, we obtained evidence to validate hydroponic saffron as a candidate for the treatment of retinal diseases. Specifically, as saffron has a positive effect on the basal cellular metabolism of ARPE19 cells [1], in this study, we tested whether this improvement was also effective in in vitro models of degenerating cells. Furthermore, we determined if these in vitro models are sensitive enough to discriminate between different types of saffron. Our results showed that the difference in saffron efficacy appears to be strongly related to the molecular composition of the saffron tested. In particular, we characterized the composition of the batches of the saffron produced using hydroponic cultures, finding that the absorbance spectra and the chemical properties (Figure 1 and Table 1) corresponded to a high-quality product (ISO standard, class I) with the same concentration of crocins that characterize the spice as REPRON^®^. We tested the protective properties of the hydroponic saffron against retinal degeneration using two in vitro models of degenerating RPE cells induced by photo-oxidative (retinol + light exposure) or oxidative (H_2_O_2_) stress [23]. The results from both models showed how saffron treatment substantially reduced cell death (Figure 3 and Figure 5). However, high-quality saffron, such as sR and sH, provide an additional support to the surviving cells by preserving the cellular cytoskeleton (Figure 4, actin) and the intercellular zone occludens (Figure 6). These discrepancies in efficiency, between reduced cell death and the maintenance of the functional morphology of surviving cells, might be related to additional, even small, differences in composition among the saffron samples, supporting the hypothesis that multidisciplinary testing is fundamental to fully unravel the neuroprotective potentiality of saffron treatment and its dependence on its chemical components, leading to coordinated and synergistic mechanisms of action. Our results confirmed that hydroponic growing is valuable for producing high-quality saffron that can maintain cell morphology and increase cell viability. Furthermore, by comparing batches with different compositions, such as sNR with the others, we found that a specific saffron composition is required not only for protecting cells from apoptosis but also for guaranteeing their physiological conditions.

## 4. Materials and Methods

### 4.1. High-Performance Liquid Chromatography (HPLC) and Spectrophotometry

We used High-Performance Liquid Chromatography (HPLC) and spectrophotometric analysis to analyze the saffron stigmas. We tested three saffron samples: S1 and S2 (hydroponic) and one field-grown sample (field). We prepared the samples for spectrophotometric analysis according to the ISO-3632 procedure [18]; however, the saffron and solvent amounts were proportionally reduced. We gently ground approximately 50 mg of saffron stigma with a mortar. We suspended 10 mg of the powdered sample in a 20 mL volumetric flask filled with 18 mL of distilled water; we maintained the suspension under magnetic stirring for 1 h in the dark, which we then finally diluted to 20 mL. The spectrophotometric measurements were obtained from a suitable aliquot of aqueous extract after a 10-fold dilution and filtration on a 0.45 μm Whatman Spartan 13/0.2 RC (Whatman, GE Healthcare Life Sciences, Little Chalfont, UK) cellulose filter. The UV–Vis spectra were acquired in the 200–600 nm range with a UV-30 Scan Onda spectrophotometer (Molecular Devices Corporate Headquarters, San Jose, CA, USA) using a 1 cm pathway quartz cuvette and pure water for blank correction. We recorded the spectra with a 1 nm resolution. We performed chromatographic analysis using Method II from a previous study [16]. We analyzed 10 μL aliquots of the saffron extracts with a Waters chromatographic system (Waters Corporation, Milford, MA, USA), consisting of a Model 600 pump, a 600 pump controller module, a 717 Plus auto-sampler, and a 996 photodiode array detector. Chromatographic data management was automated using an Empower data acquisition system (Waters S.p.A., Sesto San Giovanni MI, Italy). Eluent degassing was performed with an Agilent 1200 system (Agilent Technologies, Waldbronn, Germany). We used an analytical column (Kinetex C18. Phenomenex) with a 5 μm particle size under application of the following gradient profile: 95% A to 5% A in 30 min; 5% A to the initial composition in 5 min; and the column was re-equilibrated for 10 min. The total amount of time for the analysis was 50 min.

### 4.2. Cell Culture

ARPE-19 cells (ref. CRL-2302, ATCC Inc.) were cultured in Dulbecco′s modified Eagle′s medium/Nutrient Mixture F-12 Ham (1:1 mixture) supplemented with 10% fetal bovine serum (FBS) and 1% penicillin/streptomycin at 37 °C in a 95% O_2_ and 5% CO_2_ humidified atmosphere. The material used for cell cultures was purchased from Sigma–Aldrich (Merck, Darmstadt, Germany).

### 4.3. Cell Viability

**Figure 7:**
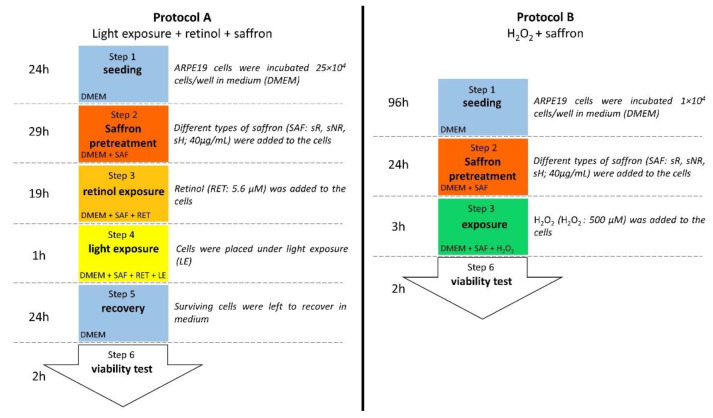
Experimental protocols. In protocol A, ARPE-19 cells were pretreated with different types of saffron (REPRON^®^ saffron (sR), non-REPRON^®^ saffron (sNR), and hydroponic saffron (sH)) for 29 h. Subsequently, cells were exposed to retinol for 19 h and then placed under light exposure for 1 h. The final viability test was performed after 24 h of recovery. In protocol B, cells were left to grow for 96 h (4 days) and were then pretreated with different types of saffron (REPRON^®^ saffron (sR), non-REPRON^®^ saffron (sNR), and hydroponic saffron (sH)) for 24 h and exposed to 500 µM H_2_O_2_ for 3 h before the viability test.

We tested cell vitality with CellTiter 96 Aqueous One Solution reagent (Promega, Madison, WI, USA) in two experimental protocols (Figure 7), where different experimental groups were compared (Table 2).

### 4.4. Light Exposure + Retinol + Saffron Protocol

ARPE-19 cells were seeded in 96-well plates (25 × 10^4^ cells/well) for 24 h. Subsequently, cells were pretreated with saffron (40 µg/mL) for 29 h, and then a retinol (R7632, Merck Inc.) half lethal dose (xhalf, 5.6 M, see Supplementary Figure S1) was added. After 20 h, cells were placed under white light (410–780 nm) from an LED 10 W–4000 K lamp (2.37 mW/cm^2^) [24] for 1 h and left to recover in the darkness for 24 h. At the end, a cell viability test (MTS-G3582, Promega srl) was performed. According to Table 2, wells were clustered in different experimental groups. REPRON^®^ saffron (sR) and non-REPRON^®^ saffron (sNR) batches were supplied by Hortus Novouls srl, whereas the hydroponic saffron (sH) was provided by ENEA spa.

### 4.5. H_2_O_2_ + Saffron Protocol

The cells were seeded in a 96-well plate at a density of 1 × 10^4^ cells/well and incubated at 37 °C in 5% CO_2_. After 4 days, cells were pretreated with 40 µg/mL of sR, sNR, or sH. The next day, we added 500 µM H_2_O_2_; after 3 h, cells were treated with One Solution Reagent and further incubated for 2 h at 37 °C in 5% CO_2_. Cell viability was normalized to the control group (no pretreatment or H_2_O_2_ exposure).

### 4.6. Immunofluorescence

We treated cells seeded into an 8-well chamber slide at a density of 1 × 10^4^ cells/well according to the protocols described above. Subsequently, cells were fixed in 2% paraformaldehyde for 15 min, permeabilized with 2.5% bovine serum albumin (BSA) and 0.3% Triton X-100 for 10 min, blocked in 2.5% BSA for 1 h, and then incubated overnight at 4 °C with primary antibodies against ZO-1 (Invitrogen, 61-7300, 1:500) and actin (mAbcam 8226, 1:200). The next day, cells were incubated with the corresponding fluorescent secondary antibodies for 2 h at room temperature. Finally, cells were counterstained with DAPI, washed three times with phosphate-buffered saline (PBS), and images were acquired using a Nikon Ni-E fluorescence microscope (Nikon Instruments Inc., Melville, NY, USA) equipped with a DS-Ri2 camera using a 20X air objective and processed via ImageJ software (Bethesda, MD, USA).

### 4.7. Statistical Analysis

We used Origin Lab 8.0 (MicroCal, Northampton, MA, USA) for data analysis and graphic presentation. All data are presented as the means ± SEMs. Statistical analyses were performed with one-way ANOVA followed by Dunnett’s post-test. A *p*-value of ≤0.05 was considered to be statistically significant.

## Figures and Tables

**Table 1:** Values of coloring (E440), bittering (E257), and aromatic (E330) power of samples (S1 and S2 are hydroponic saffron grown in a greenhouse and field-grown saffron grown using the traditional method, respectively), but only for those for which we could determine the moisture, according to ISO 3632.

Sample	E440	E257	E330	Moisture
S1	282	122	40	10%
S2	257	91	35	9%
Field Saffron	247	109	29	10%
Reference value for saffron of class 1 (dry weight) included	Greater/equal to 200	Greater/equal to 70	Between 20 and 50	Less than 12% for saffron in stigmas

**Table 2:** Experimental groups.

Experimental Groups	Tag
ARPE19 cells	Ctrl
ARPE19 cells + light exposure	LE
ARPE19 cells + light exposure + retinol	LE + RET
ARPE19 cells + light exposure + retinol + REPRON saffron	sR + LE + RET
ARPE19 cells + light exposure + retinol + non-REPRON saffron	sNR + LE + RET
ARPE19 cells + light exposure + retinol + hydroponic ENEA saffron	sH + LE + RET
ARPE19 cells + H_2_O_2_ 500 µM	H_2_O_2_ 500 µM
ARPE19 cells + H_2_O_2_ 500 µM + REPRON saffron	sR + H_2_O_2_ 500 µM
ARPE19 cells + H_2_O_2_ 500 µM + non-REPRON saffron	sNR + H_2_O_2_ 500 µM
ARPE19 cells + H_2_O_2_ 500 µM + hydroponic ENEA saffron	sH + H_2_O_2_ 500 µM

## Data Availability

The experimental data that support the figures within this paper and other findings of this study are hosted by the Department of Pharmacy, University of Pisa and Department of Physical and Chemical Sciences, University of L’Aquila, and can be accessed by contacting the corresponding author.

## Supplementary Materials

The following supporting information can be downloaded at: https://www.mdpi.com/article/10.3390/molecules28041699/s1, Figure S1: In vitro model of degenerative RPE. In less than 24 h, retinol can be internalized in ARPE cells and esterified by lecithin-retinol acyltransferase (LRAT) [24]. However, excess exogenous retinol or other retinoids followed by light exposure might induce photo-oxidation and release of superoxide radicals and subsequent cell death [25,26]. According to the corresponding protocol in Figure 1, a half-lethal dose of retinol (xhalf) was identified with a viability test with increasing retinol concentrations. The fitting curve suggests 5.6 ± 1.1 µM retinol is equal to the xhalf (A). Corresponding images describe RPE degeneration with a progressive reduction in cell volume and increasing nuclei condensation (B–I).

## Sample Availability

Samples of the compounds are not available from the authors.
