# Supplementary material for: Efficacy of Hydroponically Cultivated Saffron in the Preservation of Retinal Pigment Epithelium
Source: Molecules. 2023 Feb 10;28(4):1699. doi: 10.3390/molecules28041699 (PMC9962550; doi:10.3390/molecules28041699)
Supplement: Supplementary file 1 [file molecules-28-01699-s001.zip › molecules-2157849-SI.pdf]

Supplementary figures

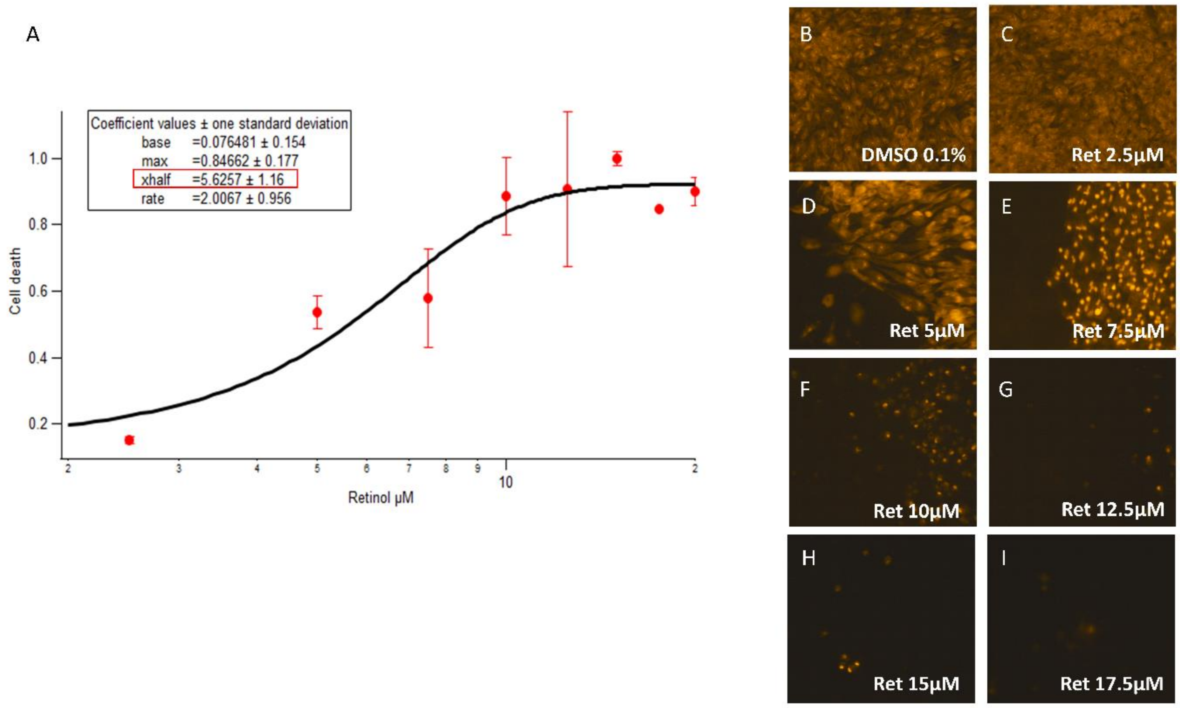

Figure S1 *In vitro model of degenerative RPE.* In less than 24 h, retinol can be internalized in ARPE cells and esterified by lecithin-retinol acyltransferase (LRAT) [24]. However, excess exogenous retinol or other retinoids followed by light exposure might induce photo-oxidation and release of superoxide radicals and subsequent cell death [25,26]. According to the corresponding protocol in figure 1, a half-lethal dose of retinol (xhalf) was identified with a viability test with increasing retinol concentrations. The fitting curve suggests 5.6 ± 1.1 µM retinol is equal to the xhalf (A). Corresponding images describe RPE degeneration with a progressive reduction in cell volume and increasing nuclei condensation (B-I).

References

24 Feldman, T.; Ostrovskiy, D.; Yakovleva, M.; Dontsov, A.; Borzenok, S.; Ostrovsky, M. Lipofuscin-Mediated Photic Stress Induces a Dark Toxic Effect on ARPE-19 Cells. *Int. J. Mol. Sci.* **2022**, *23*, 12234. https://doi.org/10.3390/ijms232012234.

25 Trevino, S.G.; Schuschereba, S.T.; Bowman, P.D.; Tsin, A. Lecithin:retinol acyltransferase in ARPE-19. *Exp. Eye Res.* **2005**, *80*, 897–900. https://doi.org/10.1016/j.exer.2005.02.013.

26 Tolleson, W.H.; Cherng, S.-H.; Xia, Q.; Boudreau, M.; Yin, J.J.; Wamer, W.G.; Howard, P.C.; Yu, H.; Fu, P.P. Photodecomposition and Phototoxicity of Natural Retinoids. *Int. J. Environ. Res. Public Health* **2005**, *2*, 147–155. https://doi.org/10.3390/ijerph2005010147.
